# Editorial: Social aspects of crop genome editing

**DOI:** 10.3389/fgeed.2025.1740380

**Published:** 2025-11-25

**Authors:** Srividhya Venkataraman, Kathleen Hefferon

**Affiliations:** 1 Independent Researcher, Toronto, ON, Canada; 2 Department of Cell and Systems Biology, University of Toronto, Toronto, ON, Canada

**Keywords:** genome editing, social impacts, regulatory issues, public acceptance, patented

While selective breeding of crops has been advocated for millennia, genome-editing enables precise, targeted and intentional changes that transcend traditional breeding practices. Obvious advantages of crop genome editing include ensuring better food security through the creation of crops having higher yields, improved nutritional profiles such as augmented mineral and vitamin content and enhanced resistance to diseases, pests as well as climate changes. Moreover, gene-edited crops can help diminish the requirement of fertilizers and pesticides, thus minimizing the environmental footprint of cultivation practices towards sustainable agriculture and conserving precious water and soil sources. Genome editing also promotes farmers’ livelihoods by reducing hazards associated with various biotic and abiotic stresses, lowering input costs and providing access to enhanced crop varieties towards providing economic viability.

However, for some of the public, the change from “natural” variation to deliberate genetic manipulation is an important ethical boundary that must be overcome. A principal concept of public perception is “naturalness”. Genome-edited crop varieties are often perceived with mistrust or rejection founded on whether they are recognized as “unnatural,” a spontaneous reaction that may misalign with scientific distinctions.

The price of research and development for generating genome-edited crops as well as their regulatory approval can exacerbate disparities within and between nations. Further, there prevail economic concerns regarding a minority of big, multinational corporations acquiring intellectual property rights and controlling the seed market. Patented seeds can incur financial encumbrance for small farmers, particularly in developing countries.

Also, there is dispute regarding whether genome-edited crop products must be labeled. Some groups call for mandatory labeling to protect consumers rights to information and opting for these products while on the other hand, other groups express anxiety that labeling could lead to rejection by the public, thus hindering innovation. Varying regulatory policies across different countries engender major challenges to international trade and promotion of new technologies. For instance, in Europe, process-based perspective on GE crops contrasts with the predominantly product-based approach adopted in Brazil, Argentina, Canada and the USA. Such differing governmental policies and not scientific reasons are the principal drivers of disparate regulatory frameworks.

Many of the world’s populace lack adequate awareness of genome editing resulting in varying and complex public perceptions. A lack of discernment can lead to mistrust in genome-edited crops with some people associating these crops with the controversial GMOs. As a result, consumers display varied attitudes towards crop genome editing ranging from approval of valuable genome-edited foods such as in Japan to profound safety concerns. Some public associations and non-governmental organizations (NGOs) actively advocate against gene-edited crops, magnifying public misconceptions and influencing policies. Public approval or rejection pose important stumbling blocks to commercialization.

This current issue of Frontiers in Genome Editing addresses the social impacts of crop genome editing in various countries such as USA, Europe, Canada, Japan and Iran highlighting their respective regulatory Research Topic as well as consumer choices. The article by Venkataraman et al. reviews the advantages of crop genome editing towards agriculture and presents examples of several organizations, industries and universities employing the genome editing technology for the development of crops. Select examples of genome edited crops having inimitable properties advancing the nutritional content of crops are presented in addition to the remarkable commercial potential of these crops. Also, the various stumbling blocks regarding the use of genome editing technology including long-term outcomes, off-target effects and other associated Research Topic are addressed. The major content of this review covers the current regulatory scenario, opportunities as well as challenges towards the adoption of genome edited crops for use by the population spanning countries such as North America and Europe in addition to the differing levels of consumer acceptance in these countries. Discrepancies pertaining to the regulatory approval schemes in several countries of the Northern part of the world will profoundly affect those of the Southern regions who have the most to benefit from these burgeoning novel technologies towards enhanced agricultural outcomes.

Similarly, “The decision to purchase genome edited food products by Iranian consumers: Theory of Planned Behavior as a social intervention tool” by Valizadeh and Karami, explored consumer attitude towards gene-edited food products. The authors found that public trust in gene-edited products and the perceived benefits of gene-edited products had positive and significant effects on the consumer behaviour and intention to purchase these products. The authors conclude that their study could provide a framework for future interventions that would improve consumer preference for food products that are genome edited.

Vasquez et al., paper entitled “Canadian Consumer Preferences Regarding Gene-Edited Food Products” explores consumer transparency and limitations in information regarding genome edited food products. The authors found four main factors which strongly influence consumer perceptions: trust in the Canadian food safety system; food technology neophobia scores; knowledge of genetics; and self-knowledge of gene editing. Survey participants indicated that nutrition, price, and taste were the three most important values. The strongest contributing factor affecting willingness to consume is the environmental impact of the production of genome edited crops. Canadian consumers largely experience more trust in genome edited rather than genetically modified crop food technologies.

While some types of crop genome editing have been quickly deregulated in Japan, societal approval of genome-edited products requires further study. The article entitled, “Consumer choices regarding genome-edited food crops: lessons from Japan” by Tetsuya Ishii investigates the sale of these products in the context of public perception through the review of the current regulatory scenario. Only a single genome-edited product, GABA tomato has been accepted as non-GMO and has been sold to consumers online catering to those with belief in its usefulness as a biofortified crop and its safety. Nevertheless, some of the consumers voice disapproval regarding their safety and demand obligate labeling. Additionally, there is lack of sufficient awareness of the technology of genome editing. Towards making informed choices of these engineered crops, it is essential to share societal perceptions, discernment of this technology and transparent labeling indicating candid information about their genome-edited identity.

In summary, the studies presented in this research topic enhance our understanding of how society is currently responding to the use of genome editing in our food system. It will be intriguing to track how things unfold from here.

